# Ribbon scanning confocal for high-speed high-resolution volume imaging of brain

**DOI:** 10.1371/journal.pone.0180486

**Published:** 2017-07-07

**Authors:** Alan M. Watson, Annika H. Rose, Gregory A. Gibson, Christina L. Gardner, Chengqun Sun, Douglas S. Reed, L. K. Metthew Lam, Claudette M. St. Croix, Peter L. Strick, William B. Klimstra, Simon C. Watkins

**Affiliations:** 1 Center for Vaccine Research, University of Pittsburgh, Pittsburgh, Pennsylvania, United States of America; 2 Department of Microbiology and Molecular Genetics, University of Pittsburgh, Pittsburgh, Pennsylvania, United States of America; 3 Department of Neurobiology, University of Pittsburgh, Pittsburgh, Pennsylvania, United States of America; 4 Center for Biologic Imaging and the Department of Cellular Biology, University of Pittsburgh, Pittsburgh, Pennsylvania, United States of America; 5 Department of Immunology, University of Pittsburgh, Pittsburgh, Pennsylvania, United States of America; Pennsylvania State Hershey College of Medicine, UNITED STATES

## Abstract

Whole-brain imaging is becoming a fundamental means of experimental insight; however, achieving subcellular resolution imagery in a reasonable time window has not been possible. We describe the first application of multicolor ribbon scanning confocal methods to collect high-resolution volume images of chemically cleared brains. We demonstrate that ribbon scanning collects images over ten times faster than conventional high speed confocal systems but with equivalent spectral and spatial resolution. Further, using this technology, we reconstruct large volumes of mouse brain infected with encephalitic alphaviruses and demonstrate that regions of the brain with abundant viral replication were inaccessible to vascular perfusion. This reveals that the destruction or collapse of large regions of brain micro vasculature may contribute to the severe disease caused by Venezuelan equine encephalitis virus. Visualization of this fundamental impact of infection would not be possible without sampling at subcellular resolution within large brain volumes.

## Introduction

Until recently, understanding cellular interaction(s) and connectivity has been hampered by an inability to contextualize interactions within the framework of the whole tissue. Images were generally collected as “representative” snapshots defined by the observer. This is particularly evident within the study of the central nervous system, which is a very large, very complex, integrated, yet compartmentalized network of cells and vasculature. At every level of resolution, from the single neuronal synapse to the anatomically isolated but interconnected functional compartment, there is a fundamental need to define complexity as a continuum such that cell development, position, interaction, and death are understood within the context of the neighboring cells, and vasculature. Recent advances in tissue clearing approaches (Sca/e[1], CLARITY[2], CUBIC[3] and DISCO (3[4], i[5], u[6]) have allowed imaging at great depths, making the third dimension obtainable and enabling imaging of whole brains.

Once cleared, the most time limiting step to collecting high resolution confocal images has been the need to collect single two dimensional snapshots using a stop-and-shoot approach in which the stage is moved, the scanning is initiated, the image is collected and the process is repeated followed by image stitching. Even when utilizing confocals with fast resonant scan heads the process remains slow and takes 2–5 seconds between positions. Commonly, complex multipanel images are stitched on 3 axes which takes significant time following collection. To satisfy Nyquist sampling, as resolution increases, the number of fields are essentially cubed according to the change in magnification and Z sampling, compounding the time to acquire high-resolution volumes. Light sheet methods, in which the sample is illuminated laterally with a thin sheet(s) of light, allows rapid collection using sCMOS detectors. Generally, a relatively low resolution overview is acquired which is used to guide collection of a higher resolution subimage, with a different objective or intermediate zoom optics (for example the LaVision system). However, this method demands complex sample mounting approaches and minimization of the refractive index change across the sample. Ultimately, there is a significant compromise in Z-resolution due to the inherent and somewhat variable thickness of the illuminating light sheet, and the XY resolution is limited to a circumscribed choice of collection optics.

In this paper, we report the application of a recently developed confocal approach which does not depend on the traditional “stop-and-shoot” approach. The system uses a resonant single axis scanhead coupled to a fast stage which scans “ribbons” in a single movement. This allows large areas to be imaged in multiple colors very rapidly, collecting a 40,000*40,000 pixel mosaic image in under 3 minutes as opposed to the 1.5 hours required using conventional confocal imaging. As stitching only occurs on a single axis, stitching artifacts are minimized and stitching speed is dramatically increased. The ultimate resolution using this method is exactly equivalent to other confocal methods, limited only by the wavelength of the light used and the numeric aperture (NA) of the illumination/collection objective. We demonstrate the viability and speed of the solution for scanning large areas of tissue (marmoset brain) in multiple colors. Furthermore, we find that the sensitivity and three axis resolution of the device allows us to detect single infected neurons within an entire mouse brain following exposure to the encephalitic Venezuelan (VEEV), eastern (EEEV) and western (WEEV) equine encephalitis viruses. At a late time point in infection with VEEV, microvasculature surrounding regions of heavy virus replication was destroyed or inaccessible, a novel observation facilitated by ribbon scanning confocal imaging of chemically cleared brains. While we are using this approach to gain insights into viral pathogenesis, its potential within imaging of all cleared tissue samples is revolutionary when compared to other currently available technologies.

## Materials and methods

### Ethics statement

All animal procedures were carried out in accordance with University of Pittsburgh Institutional Animal Care and Use Committee guidelines under the approved protocols 12100628 and 15076006 for rabies and alphavirus studies, respectively.

### Animals and virus infections

Three-year-old male common marmosets (*Callithrix Jacchus*) were obtained from Wisconsin National Primate Research Center. The Marmosets were housed in an AAALACi accredited facility in Britz & Co. cages with approximately 4.2 sq ft cage space per quad. The cages are structurally enhanced with multi-level shelving and ladders, hammocks, and hanging toys. A variety of enrichment was provided weekly including cardboard boxes, cups, aspen bedding, and tunnels of varying sizes. Animals were pair housed or housed in family groups to provide social enrichment. Housing rooms were maintained at 75–78 degrees F and a humidity range of 30–70%. Animals were fed a standard biscuit diet from Mazuri (Callitrichid Gel Diet # 5M16) with Vitamin D3 added. Fruits, vegetables, greek yogurt, acacia gum and protein supplements (crickets, mealworms and waxworms) were fed in addition to the standard diet. Marmoset were infected with the N2c strain of Rabies Virus (Matthias Schnell, Thomas Jefferson University) by injection in to the extensor digitorum communis muscle. The animals were monitored at least twice daily prior to surgery and every 4–8 hours following surgery. During surgery, marmosets were anesthetized via inhalation of isofluorane 1–3% (vol/vol) with up to 5% for induction. On hour 117 following infection, euthanasia was carried out by anesthetizing the animals with isofluorane (3–5%) and then administering pentobarbitol (40mg/kg) IP. The animals were then administered three solutions by transcardial perfusion: (i) 0.1M phosphate buffer (pH 7.4), (ii) phosphate-buffered formalin (3.7 g of formaldehyde gas/100 ml of 0.1 M phosphate buffer), and (iii) a solution of 10% (vol/vol) glycerol in phosphate-buffered formalin at 4°C. 250-400ml of each solution. None of the marmosets showed severe signs of illness following infection or died without euthanasia.

Wistar rats were obtained from Charles River. Rats were housed at 68–79 degrees F, 30–70% relative humidity, 12-hour light/dark cycle and fed a commercially available diet. The rats were infected with the N2c strain of Rabies Virus in the whisker vibrissa. On day 4.5 following infection, the animals were deeply anesthetized and then sacrificed by transcardial perfusion by PBS then phosphate buffered 10% formalin. The brain was then extracted and fixed in phosphate buffered 10% formalin for 3–5 days prior to sectioning.

CD1 Mice were ordered at six weeks of age from Charles River and were housed under the same environmental conditions as rats. The mice were infected at ABSL-3 within the University of Pittsburgh Regional Biocontainment Laboratory. Virus was introduced subcutaneously in a single rear footpad with 1e03 PFU of Venezuelan equine encephalitis virus (VEEV) expressing mCherry. mCherry was inserted into the V3000 of the Trinidad Donkey strain of VEEV (VEEV TrD TaV-cherry) as previously described [7]. GFP was inserted into the western equine encephalitis Virus (WEEV) McMillian stain (WEEV McM TaV-GFP) as previously described [7]. Aerosol infections were completed with WEEV McM TaV-GFP and the FL93-939 strain of eastern equine encephalitis virus expressing GFP (EEEV TaV-GFP) [7]. Virus was produced from infectious clones and tittered as previously described [7]. Aerosol exposure of mice was conducted using the AeroMP (Biaera technologies, Hagerstown, MD) [8]. Mice were euthanized at 72–96 hours post infection and perfused transcardially with PBS and then 4% PFA or 4% PFA containing 20nm yellow-green fluorescent beads (Thermo Fisher, F8787). Brains were fixed in 4% PFA at 4 degrees Celsius for 24 hours prior to removal from ABSL-3 containment.

### Marmoset and rat brain tissue preparation

Marmoset brains were sectioned using a vibratome into slices of 50μm thickness. Tissue sections were washed in PBS 3 times for 10 minutes each at room temperature. They were then blocked in a solution of 0.5% TritonX-100, 4% Normal Goat Serum (NGS) in PBS for 2 hours at room temperature. Following blocking, primary antibody was applied in a solution of 1% NGS, Rabbit anti-NeuN (Millipore 1:500), Mouse anti-Rabies (31G10[9] 1:50), and PBS for 48 hours at 4 degrees. The sections were again washed in PBS 3 times for 10 minutes each at room temperature. Next they were treated with secondary antibody in a solution of 3% NGS, 0.3% TritonX-100, Goat anti-Mouse IgG1 Alexa 488 (1:500), Goat anti-Rabbit IgG Alexa 647 and PBS for 2 hours at room temperature. Following secondary antibody, the tissue slices were washed again in PBS 3 times for 10 minutes at room temperature. They were then mounted on slides and allowed to dry. When dry, the slides were rinsed with ddH_2_O and again allowed to dry. The slices were then coverslipped with cytoseal.

### Mouse brain tissue preparation and CUBIC clearing

Mouse brains were incubated overnight at 4 degrees Celsius in hydrogel solution[2] in DPBS and without PFA (4% acrylamide, 0.05% bis-acrylamide, 0.25% VA-044). The hydrogel was polymerized by shifting the brains to 37 degrees Celsius for 4 hours. Brains were then horizontally sectioned with a razor blade to a thickness of approximately 4mm to include the olfactory bulb. The sections were cleared using CUBIC [10] by immersion in 50% CUBIC R1 for 1 day, then the solution was changed to 100% CUBIC R1 on days 2, 3, 7 and 10. Incubations were completed at 37 degrees Celsius with gentle movement. The cleared tissue was mounted in CUBIC R1 for imaging.

### Microscopy

Ribbon scanning microscopy was completed on the pre-commercial RS-G4 confocal microscope purchased from Caliber I.D. Large-area mosaic images were captured using either the ribbon-scanning or frame modes. Laser powers used for imaging varied according to each tissue and the depth of each optical section but were approximately 1–2% for 50μm sections of marmoset brains or 15–30% for deep tissue imaging of mouse brains. Most volumes were acquired with z-steps of 25.15μm to impose practical limits on the scale of the data collected (under these conditions, our largest dataset was approximately 750 gigabytes). Lateral per-pixel resolution was dependent on the objective: 0.551μm (Olympus 25x, 1.05NA, water), 0.722μm (Nikon 20x, 0.95NA, water), 0.740μm (Nikon 20x, 0.45NA, ELWD, air). Z-steps of 2.28μm with a lateral per-pixel resolution of 1.430μm (Nikon 10x, 0.5NA, 5.5WD, glycerol) was used to acquire the two-color volume from S6 Movie.

The resonant scanner frequency was 8000Hz with a pixel dwell time of between 30 and 100 nanoseconds. Two Hamamatsu photo multiplier tube detectors were used to detect emitted light from laser lines 488 (H13175U -110), 561 and 647 (H13175U -20). The pinhole size was 75μm (488) or 60μm (561, 647). Image frames were collected at 1024 x 1024 pixels at a rate of 5.9 frames per second. A Marzhauser Scanning stage SCANplus IM 120 x 80 (00-24-579-0000) was used to continuously move the samples under the scan head during ribbon acquisition. Z-alignment was based on the stage mechanics which has an accuracy of 1μm and a minimum step size of 0.05μm. For a stack, the stitch was calculated on the middle layer and applied to each subsequent layer, ensuring that each z-plane was assembled in precisely the same manner. Due to the speed of image collection, custom designed workstations were built for acquisition that included 64GB of RAM and four 1TB solid-state drives configured in RAID0.

Beyond the ribbon scanning system, the confocal microscopes used in this study were a Nikon A1, A1R and Ti equipped with a Bruker (Bruker Scientific, Madison Wi) Sweptfield Scan Head (now Opterra). In each case the images were captured with a 20x, 0.75NA object. In the case of the Nikon A1 images were collected with a 250nm point spacing, with the A1R a 512x512 resonant image was collected with a zoom of 1x, with the sweptfield scan head images were collected using a 512x512 Andor (Belfast Ireland) Ultra EMCCD camera, 50ms exposure time, 1x zoom. The multiphoton volume acquired for this study used a Nikon A1 multiphoton, galvano line scanner with 2x averaging, 3μm z-steps.

### Image Reconstruction

Two dimensional images of marmoset brains were adjusted for background and contrast using Nikon Elements.

Volume data sets ranged in size from 150-850GB. Data storage and processing infrastructure was assembled for this project that included:

1. A high-speed file server with 160TB of raw storage capable of delivering 10 gigabits/s.
2. A computational server with dual Intel Xeon E5-2630 v3 processors, 512GB of RAM, dual NVIDIA GeForce GTX 1080 graphics and 32TB of solid-state drive storage configured in RAID0.
3. Numerous workstations were constructed that included an Intel i7-6850K processor, 128GB of RAM, NVIDIA GeForce GTX 1080 graphics, and 1TB of solid-state storage.
4. 10 gigabit networking was distributed to all systems.

Mosaic images were assembled into volumes using Imaris File Converter. The volumes were manipulated in Imaris v4.0, configured to utilize 75% of the available RAM per workstation with the cache directory pointed to solid-state storage. Large volumes that required significant computational resources, for example the joining of the two independently imaged volumes, were completed on the computational server with Imaris configured to use 450GB of RAM and the cache configured to use the 36TB RAID0 solid-state array.

## Results

### Ribbon scanning confocal microscopy is a high-speed high-resolution means of acquiring large area mosaics

**Fig 1:**
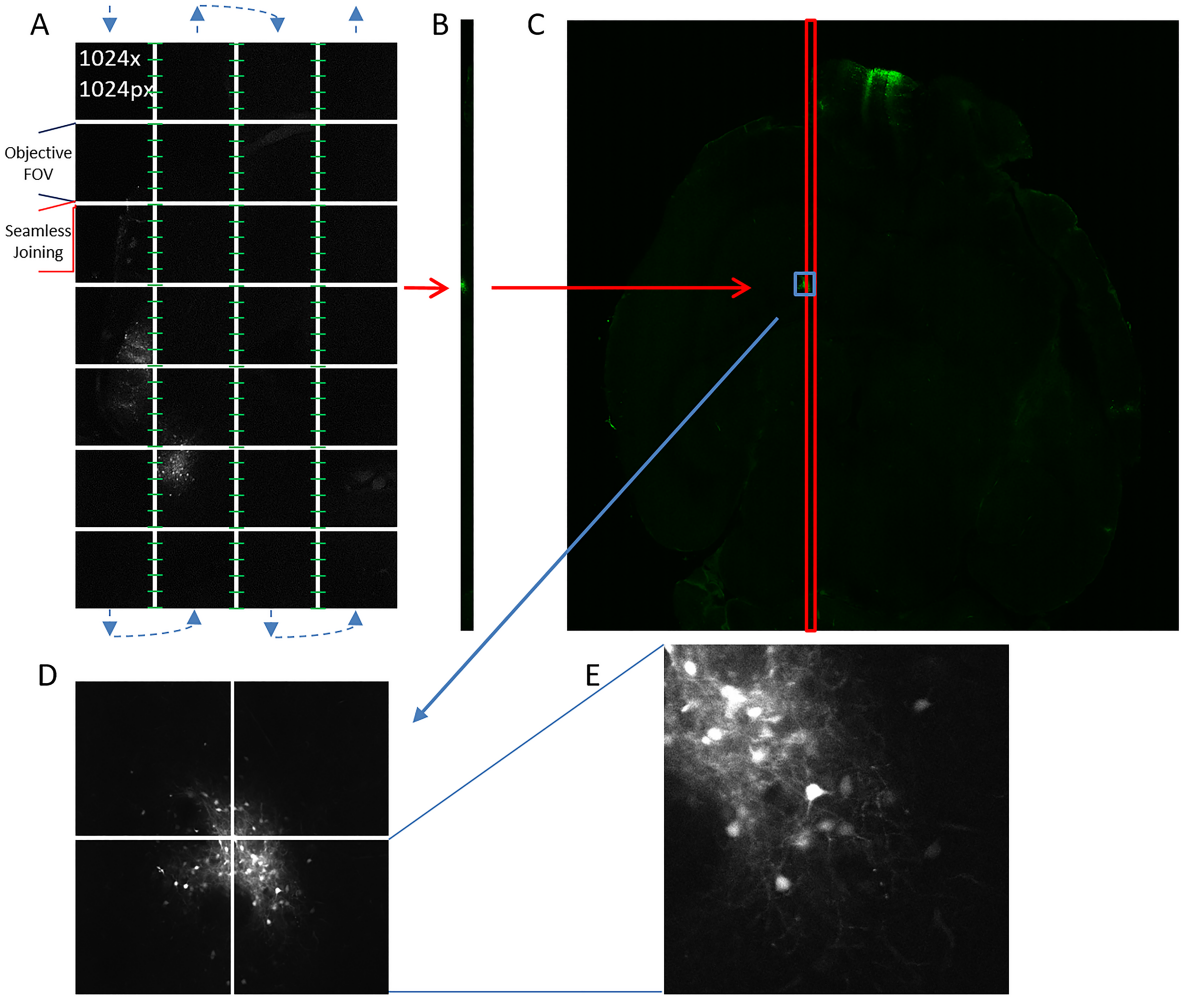
The ribbon scanning approach. (A) The blue arrows indicate the direction that the sample moves under the objective, acquiring non-overlapping individual fields of view, and pausing only at the end of each ribbon to advance on to the origin of the next ribbon. Each ribbon overlaps by ten percent with its adjacent ribbon. The overlap is used to stitch (green lines) each ribbon following its completion. (B) Completed ribbons are assembled into a (C) composite image that is representative of a single large-area scan. (D) Multiple fields of view elucidate regions or structures of interest within a larger area whereas each (E) individual field of view contains subcellular detail.

Traditional large area mosaic imaging systems utilize a ‘stop-and-shoot’ modality that requires that sample stage to be precisely positioned for each tile. This requires the initiation of mechanics that move the sample and actuate changes in shutter and laser positions for every tile within the mosaic. The sample is positioned so that each tile within the mosaic overlaps with contiguous tiles for computational joining (i.e. stitching) to form a single image. The oversampling required to collect this overlap can constitute 20–30 percent of the total image. In contrast to traditional ‘stop-and-shoot’ mosaic imaging systems, ribbon scanning confocal microscopy uses a continuous stage movement timed precisely to a high-speed resonance scanner (Fig 1A). The microscope acquires in vertical ‘ribbons’ where each image within the ribbon, comprised of a single FOVs, joins seamlessly (e.g. no overlap) along the x-axis. The absence of overlap within a ribbon eliminates the requirements for over sampling and the computational overhead required for stitching. During acquisition, the stage moves in a serpentine pattern under the scanhead, pausing only momentarily at the completion of each ribbon to advance on the x-axis to the origin of a new ribbon. Each ribbon is acquired with overlap on one side resulting in an oversampling rate of approximately ten percent. With the completion of each ribbon (Fig 1B), the software initiates on-the-fly stitching with the adjacent ribbon which results in the near real time assembly of the final mosaic image (Fig 1C). When paired with a low magnification high NA objective (25x, 1.05NA), a single z-plane from a CUBIC cleared mouse brain was completed in under three minutes (Fig 1C). Each tile in the final mosaic represented one field of view from the objective (Fig 1D) and resolved subcellular detail from cells infected with a GFP-expressing virus (Fig 1E).

### Ribbon scanning of large area samples is faster than conventional high-speed confocal microscopy with equivalent quality and reduced stitching artifacts

**Fig 2:**
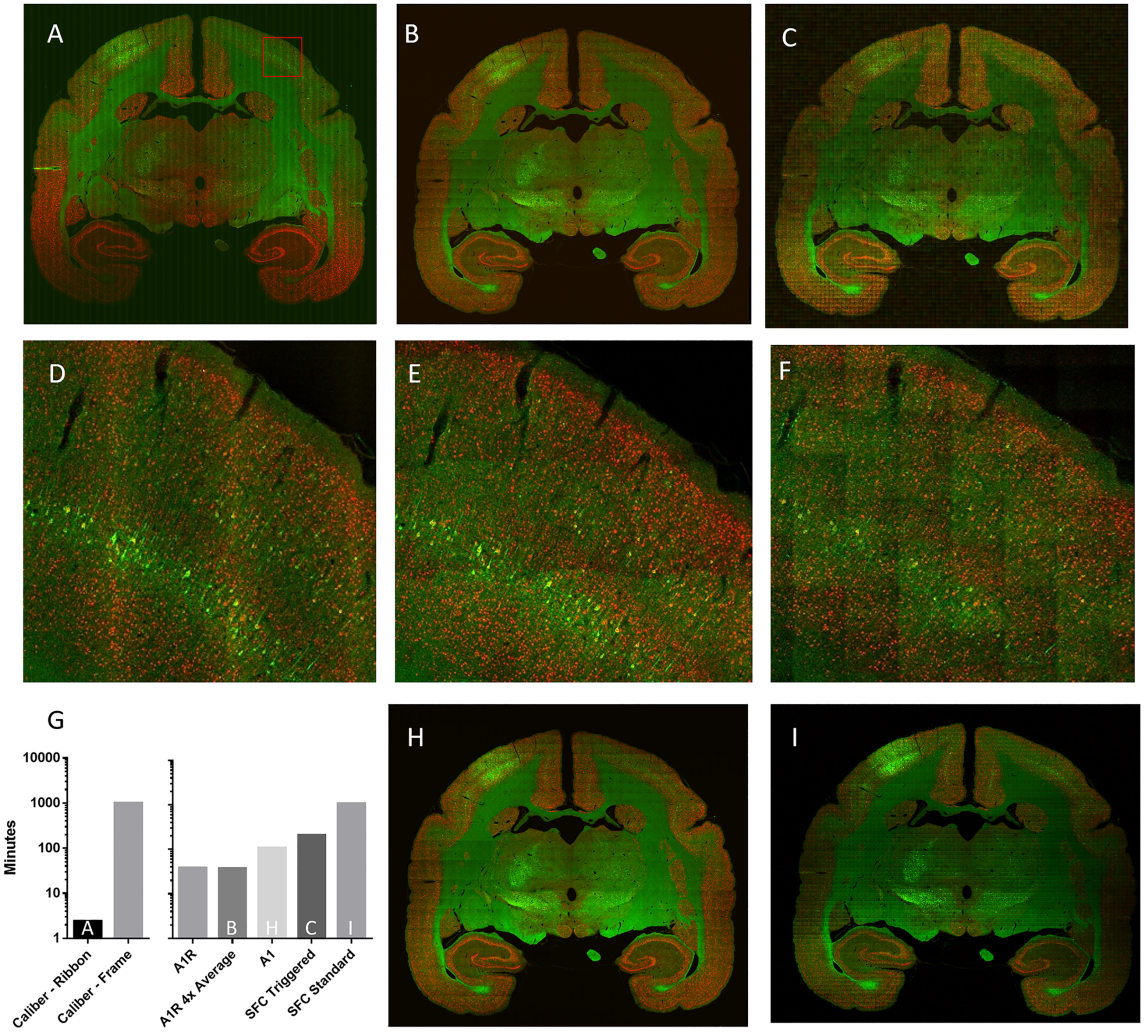
Ribbon scanning confocal microscopy is faster than conventional confocal microscopy and produces equivalent quality and reduced stitch artifacts. A single 10x10 millimeter dry mount coronal section of rat brain was imaged on (A) the Caliber I.D. ribbon scanning confocal (Olympus 20x, 0.7NA) (B) the Nikon A1R using 4x averaging (Nikon 20x, 0.75NA) and (C) a swept field confocal using high-speed triggered acquisition (Nikon 20x, 0.75NA). Panels D-F are zoomed regions from panel A-C, respectively. The zoomed regions correspond to the red box in panel A. The times of acquisition for various microscopes and modes is recorded in panel G. Acquisition on a (H) Nikon A1 using a Galvano line scanner or (I) swept field confocal using standard sequential acquisition with line averaging led to better quality than the corresponding panels B and C but with additional time penalties.

We compared the speed of ribbon scanning and commercial confocal microscopes for imaging large areas. Marmosets were infected with rabies virus and coronal sections were stained for virus proteins and nuclei. Using equivalent objectives, a 10x10mm area from a 50μm section was acquired as a single optical plane mosaic by ribbon scanning (Caliber I.D., Fig 2A), a Nikon A1R (Fig 2B) and a high-speed Nikon swept field confocal (Fig 2C). All methods of image acquisition resulted in similar quality (Fig 2D–2F) effectively resolving sub cellular details including cell bodies, apical and basal dendrites, along with dendritic spines of infected cells. The sub cellular detail was improved with ribbon scanning when viewed through a higher magnification objective (S1 Fig). Ribbon scanning resulted in an improvement in the overall quality of the mosaic due to the reduced number of stitch seams (Fig 2A). The ribbon scanning methodology improved the speed of acquisition by 413-fold compared to frame-by-frame based acquisition on the same instrument (Fig 2G). The overall time to completion of a single mosaic was under 3 minutes by the ribbon scanning approach whereas the fastest comparable instruments, the Nikon A1R, Nikon A1 and Nikon swept field confocal (SFC) microscopes completed in 37–38 (Nikon A1R), 108 (Nikon A1), 210 (SFC triggered simultaneous acquisition) and 1049 minutes (SFC standard sequential acquisition, line averaging) (Fig 2G). The quality of the images from the Nikon A1R and SFC microscopes were improved by using the Nikon A1 which uses a galvano scanner (Fig 2H) or line averaging on the SFC (Fig 2I), features not available on the Caliber I.D. ribbon scanning confocal. However, these methods of acquisition further increased the time of acquisition (Fig 2G).

### Depth imaging in chemically cleared brains is superior by confocal when compared with multiphoton

**Fig 3:**
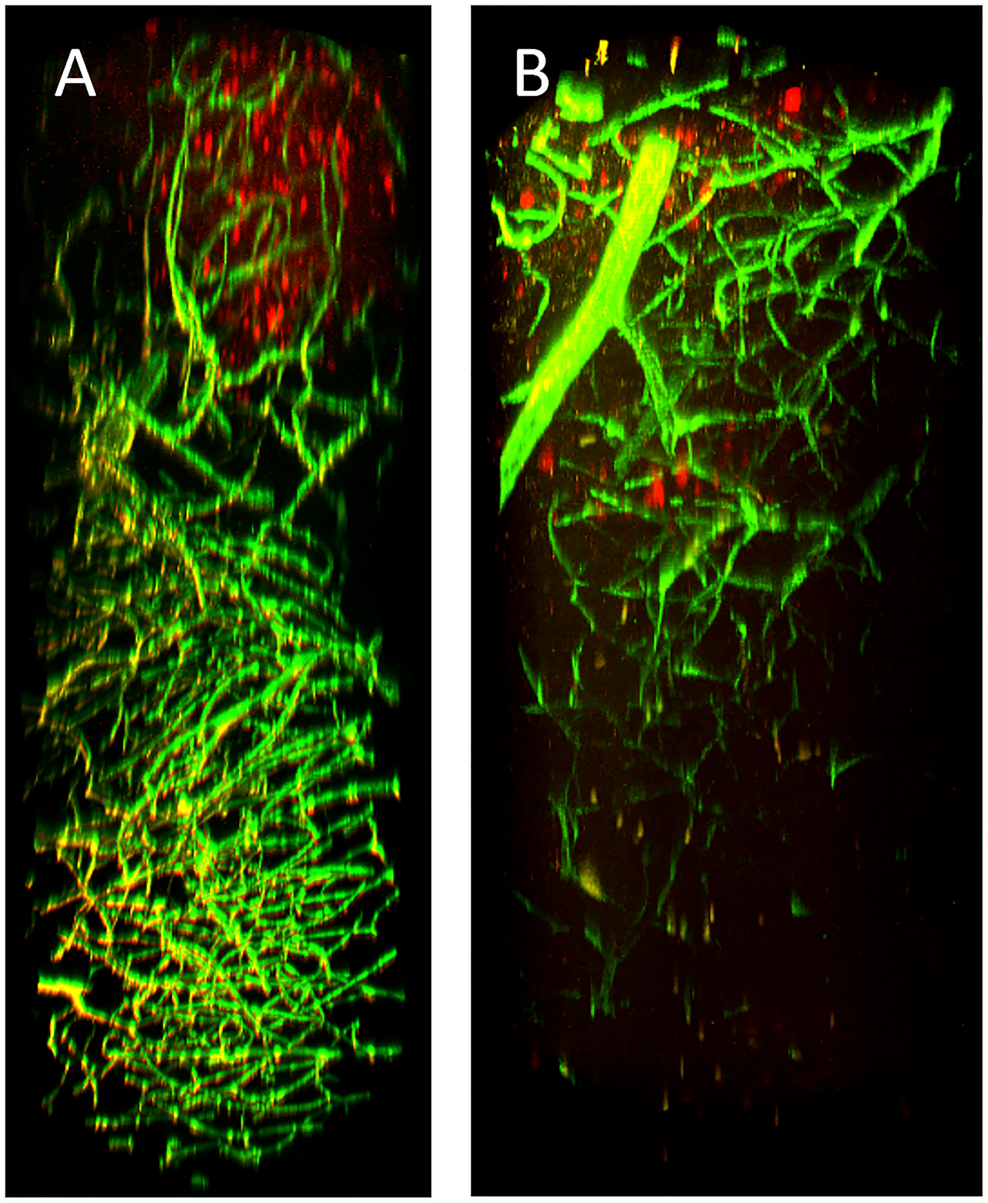
Confocal is more effective than multiphoton at multicolor deep tissue imaging in chemically cleared brains. A mouse was infected subcutaneously with VEEV TrD TaV-cherry (red) and at 96 hours post infection, fluorescent beads (green) were introduced into the vasculature by cardiac perfusion. The brain was harvested, sectioned approximately four millimeters thick, and cleared by CUBIC prior to imaging on the (A) Caliber I.D. ribbon scanning confocal and a (B) Nikon multiphoton. An Olympus 25x, 1.05NA, 2.0WD objective was used to capture both volumes.

Large area acquisition is most useful in the context of deep volume imaging where whole tissues can be reconstructed in three dimensions. Depth imaging has historically been the domain of multiphoton microscopy which utilizes red-shifted ultrafast pulsed lasers and long working distance objectives to enhance tissue penetration. Conversely, depth penetration using conventional confocal imaging is limited to about 50 microns, due to a lack of light penetration into the tissue and a failure of the emitted light to pass through the confocal pinhole caused by scattering and absorbance. However, when either single or multiphoton systems are paired with tissue clearing protocols that match the refractive index of the tissue and remove light-refracting and light-absorbing molecules like lipids and chromophores[1–6], imaging can be extended to multiple millimeter depths. When cleared tissue is used, the standard confocal microscope is generally more facile to use than multiphoton methods. The most compelling reason is the ability to image multiple concurrent channels with a broad spectrum of specific wavelengths. Using a long working distance multiphoton objective designed for deep tissue imaging (25x, 1.05NA, 2.0WD), we compared imaging in depth with the Caliber I.D. confocal (Fig 3A) and a multiphoton (Fig 3B) microscope. We found that, with tissue clearing, both methods allowed for imaging of cleared brain tissue at depths of two millimeters (the limit of the objective). In contrast to the multiphoton excitation, the Caliber I.D. confocal was more effective at distinguishing signal deep in the tissue. The cross-talk observed between the 488 and 561 channels using the Caliber I.D. system was the result of simultaneous scanning, which is analogous to multiphoton where light is collected simultaneously. Simultaneous scanning was used for two-color ribbon-scanning in this study since the ribbon-scanning confocal experiences no change in the rate of acquisition while ribbon scanning. S2 Fig demonstrates that, like other confocal systems, sequential imaging reduces cross-talk between these channels.

### Ribbon scanning confocal microscopy facilitates rapid high-resolution imaging of chemically cleared deep brain tissue

**Fig 4:**
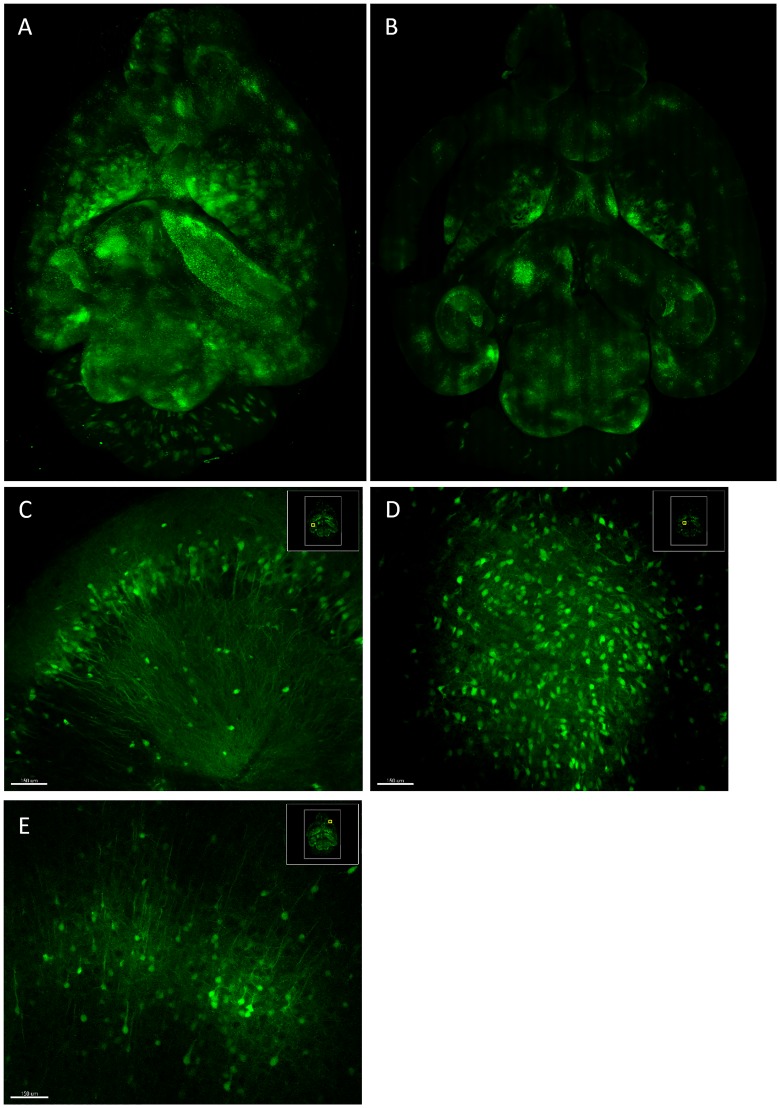
Ribbon scanning confocal microscopy captures subcellular detail throughout large volumes in chemically cleared mouse brains. A mouse was infected by aerosol with EEEV TaV-GFP. At 96 hours post infection, the brain was harvested, sectioned approximately four millimeters thick, and cleared by CUBIC. The Caliber I.D. ribbon scanning confocal was used to reconstruct a (A) 21.0 x 15.5 x 1.735 millimeter volume. (B) A single optical plane from the volume demonstrates the (C-D) subcellular detail captured throughout the volume.

We then used the Caliber I.D. system to image through a cleared brain from a mouse infected with a GFP expressing alphavirus. The brain was embedded in hydrogel and coarsely sectioned to approximately 4mm thick prior to clearing by CUBIC. The area of each optical section was 21.0 x 15.5 millimeters acquired in z at 25.15 micron intervals. The stitch for each z-plane was determined based on the middle layer, which was acquired first, and that stitch was subsequently applied to all other layers. The z-alignment is demonstrated in S1 Movie. The resulting 3D reconstruction (Fig 4A) took three hours and twenty minutes to complete and spanned 1735 microns in depth, approximately 1/4 of the mouse brain. Each optical plane (Fig 4B) was acquired in 2.9 minutes with a resolution of approximately 551nm per pixel. Infected neurons were captured in subcellular detail throughout the volume (Fig 4C–4E; S2 Movie), evidenced by the visualization of axons projecting from individually infected cells.

**Fig 5:**
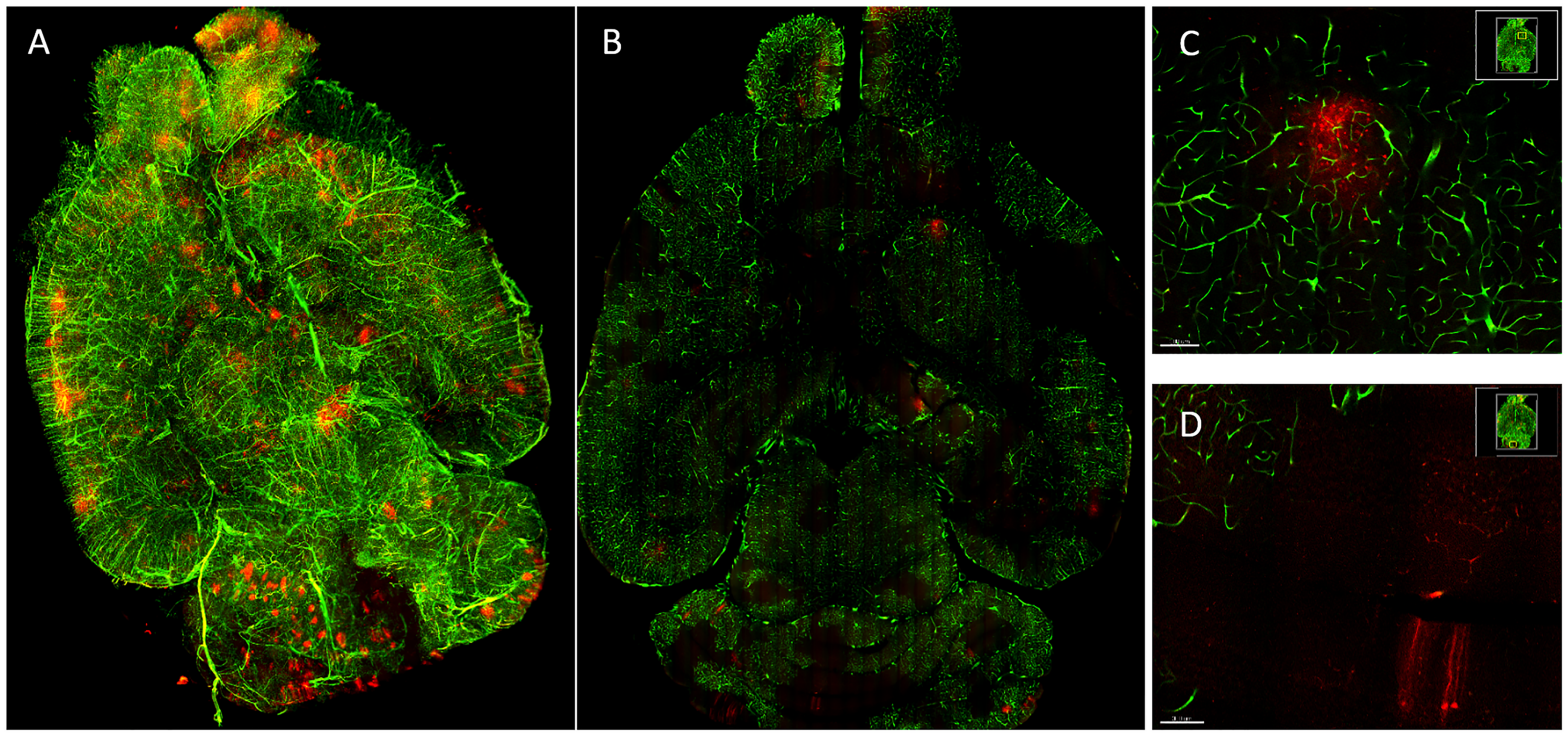
Multicolor large-volume imaging by ribbon scanning confocal microscopy. A mouse was infected subcutaneously with VEEV TrD TaV-cherry (red) and at 96 hours post infection, fluorescent beads (green) were introduced into the vasculature by perfusion. The brain was harvested, sectioned approximately 4mm thick, and cleared by CUBIC. The section was imaged approximately two millimeters deep (the limit of the Olympus 25x, 1.05NA objective) on both sides and reconstructed as (A) one volume. (B) A single optical plane demonstrates that both the microvasculature and virus infected cells were observed throughout the volume. (C-D) Zoomed regions from a single optical place demonstrate that the vasculature in some areas with virus replication were inaccessible to fluorescent beads administered by perfusion.

We then tested the utility of ribbon scanning for imaging large volumes and in multiple colors. A mouse was infected with a VEEV mCherry expressing virus and at the time of harvest we introduced green fluorescent beads into the vasculature by perfusion. Using the long working distance multiphoton objective, we imaged through one side of the section and then imaged again from the reverse side. Using simultaneous two color scanning, each optical section took 3.8 minutes to image, a total of 9.7 hours for both sides. The two images were joined into a single reconstructed volume representing approximately ½ of the mouse brain (4174 microns of tissue (Fig 5A, S3 Movie)). Each optical section displayed the full microvasculature within the tissue (Fig 5B) as well as individually infected cells at subcellular resolution (Fig 5C and 5D). We found that in many regions with strong virus replication, the microvasculature did not exhibit signal from the beads (Fig 5D, S3 Movie), a novel observation suggesting that virus replication may result in vascular destruction or occlusion.

High NA multiphoton objectives are expensive and are commonly difficult to obtain without the purchase of a concomitant multiphoton system. To demonstrate that large volume imaging by ribbon scanning is plausible using more accessible objectives, we imaged the previous sample with a water immersion Nikon objective (20x, 0.95NA, 1.0WD) (S4 Movie) and a dry ELWD Nikon (20x, 0.45NA, 7.4WD) (S5 Movie). While there were obvious compromises in depth penetration and signal recovery, due to working distance limitations or reduced NA, image quality remained high, and the resolution was sufficient to identify individual virus-infected cells and microvasculature. The working distance of the water immersion Nikon objective limited depth imaging to half that of the multiphoton objective and decreased the lateral resolution by 25% (722nm) per pixel, but the detail captured from this objective was visually comparable to the multiphoton objective including excellent signal recovery in deep tissue and subcellular and microvasculature detail. The ELWD Nikon 20x air objective is similar to those used on light-sheet systems capable of imaging whole mouse brains. Using this objective, we were able to acquire the whole tissue section in a single imaging session, requiring no computation to reconstruct the whole tissue as was required with the multiphoton lens in Fig 5. The resulting images had a resolution similar to the 20x water lens (740nm per pixel), but signal originating from deep in the tissue appeared weak.

Finally, we used a new 10x, 0.5NA, 5.5WD objective designed by Nikon to acquire a sub-volume of a brain infected with Western Equine Encephalitis virus at a depth of 4.2 millimeters, 2.28 microns per slice (1850 z-slices) and 1430nm per pixel resolution. Individually infected neurons and the surrounding micro vasculature are visible throughout the volume (S6 Movie). The volume represents approximately ¼ of a whole brain and required 17.7 hours to acquire at 30 seconds per optical slice. These data suggest that low magnification, high NA, long working distance objectives can be paired with ribbon scanning to image whole brains in under two days.

## Discussion

Here we demonstrate the potential of ribbon scanning confocal microscopy to rapidly image large-area tissue sections of marmoset brains and reconstruct large-volumes of chemically cleared mouse brains at subcellular resolution. This approach increases the speed of large-area / large-volume confocal microscopy by at least a factor of 10 when compared to commercially available systems. When paired with chemical clearing of tissues, the approach makes the collection of truly high-resolution large-volumes feasible. The novel combination of chemically cleared brains, confocal ribbon scanning and high-NA long working distance objectives allowed us to sample one-half of a brain in nine hours. We also demonstrate a novel use for this technology to study the pathogenesis of encephalitic alphaviruses. We observed the breakdown or occlusion of microvasculature in large regions of the brain associated with advanced virus replication, an observation that would not have been feasible using traditional confocal imaging approaches. We suggest that the speed and utility of this approach (when applied to the brain and other organs) makes it broadly applicable to the study of infectious disease, gene expression, developmental biology and neurobiology.

The power and capability of microscope imaging tools has grown exponentially over the last 10 years. This has been driven by advances in the four essential components of the technology; molecular probes, optics, robotics and computing. Coincident with these advances has been a reinvention of the field such that it has moved from a set of confirmatory and principally descriptive tools to a primary method of investigating scientific problems. Along with major advances in live cell, live animal and super-resolution imaging there has been a drive to collect more complete data and ensure that quantitation is robust and reproducible. Historically scientists have prepared thin sections of fixed tissue and then used collection and rendering tools to generate 3D images of neuronal pathways from many thin sections. This approach has been in place since the days of Ramon Y Cajal [11] and continues to the present day using optical sectioning approaches (either confocal of multiphoton microscopy). While confocal methods allow exquisite reconstructions of small tissue regions, extending the method to the whole brain has proven quite difficult and extremely time consuming. Early efforts used multiphoton methods which allowed significantly deeper imaging (up to about 500μm) into tissue because of the selective plane illumination, the longer wavelength light and the absence of a pinhole. More recent advances in chemical clearing [12] of thick sections, even whole brain, have allowed single photon imaging at depths of many millimeters.

Collecting large volume data by confocal microscopy has been onerous and extremely time consuming. Our results (Fig 2G) suggest that, using a galvanometer driven scanhead, individual images take about 4 seconds/tile (1024*1024 pixels) to collect such that a simple 10mm*10mm*100slices (XYZ) image would take approximately 45 hours (1.9 days). Resonant scan heads increase this speed significantly to about 14 hours (0.6 days), approximately 3 fold in our hands. We show that an additional and dramatic decrease in speed is possible through ribbon scanning such that the same image would take only 1 hour.

To date, rapid whole brain imaging has generally been the domain of light-sheet microscopy. Light-sheet systems provide numerous benefits over traditional confocal systems that result from the lateral illumination of only a single optical plane [13]. The mode of illumination and the use of fast cameras results in the rapid capture of large volumes without the need for point-scanning during acquisition and reduced photobleaching/photodamage. However, light sheet systems require specialized mounting schemes that allow for both lateral illumination and axial collection of light making sample preparation and mounting arduous. Using the ribbon scanning confocal imaging technology described in this study, we have imaged tissues mounted on traditional glass slides or whole tissues in coverslip covered sealed volumes of clearing fluid. This seemingly simple detail allows for a sample to be interrogated using multiple imaging modalities without the need to remount and reposition a sample.

If the refractive indices of tissue and clearing fluid are not perfectly optimized through a sample preparation, illuminating light-sheets suffer from both diffraction and refraction within tissues. Also, unless multiple rotational points are collected and integrated or multiple sheets are used there is a degradation in image quality moving across the sample. In the ribbon scanning system, the optical sectioning is a function of the pinhole which limits the collection out-of-focus light and the axial resolution is controlled by the specific objective. These properties make the ribbon scanning confocal relatively tolerant of refractive index mismatches thus providing higher resolution images deep in tissue. This approach also allows the user to pull from the complete array of objectives already developed for confocal microscopy, and the choice of objective is constrained only by the experimental needs (e.g. working distance, resolution, sample brightness), not by the physical constraints of the imaging platform.

A primary concern with imaging large volumes is reducing photobleaching and photodamage over the long acquisition times. Ribbon scanning confocal uses a combination of a high-speed resonance scanner and image acquisition rate to reduce exposure of the sample to excess light. Each pixel is interrogated by the laser for a maximum of 100ns. For the large multicolor volume that was imaged in this study, each region of the tissue was exposed to intense laser interrogation for only an accumulated 15μs (30μs for the regions where ribbons overlap). We imaged the same volume multiple times with no appreciable diminution in fluorescence activity. Importantly, high resolution reconstruction of a whole brain volume could require an order of magnitude more optical slices to complete than were demonstrated in this study. Thus, the extrapolated exposure for 3000 slices would be only 300μs (600μs for regions of overlap) of laser interrogation for any given region of the tissue.

With the advent of ribbon scanning technology, the rapid collection of large area confocal data sets is now accessible. Ribbon scanning technology is as high-resolution and flexible as confocal imaging systems with the speed of wide field imaging systems that are used on light-sheet microscopes. This combination is a powerful tool that enables the more complete collection of large-area / large-volume data sets that could range from live cells to whole animals on a single platform. We expect that the ribbon scanning confocal system will be more cost effective for researchers since the speed of acquisition will enable the collection of multiple whole tissues or organisms in a single day. Additionally, we expect that a complete ribbon scanning system (once commercially available) is likely to be 3–4 time less than currently available light-sheet solutions. The expected affordability coupled with the rapid acquisition of data will elevate subcellular imaging of whole tissues and organisms to a principal means of experimental evaluation and perhaps into the toolbox of fields unrelated to neuroscience.

## Supporting information

S1 Fig Ribbon scanning demonstrates sub cellular detail of a complete rat coronal section using 40x magnification. (A) A whole coronal section of a rabies infected rat brain was stained for rabies (green) and nuclei (NeuN, red). (B) A zoomed imaged from panel A with the location designated by the red box. The image was acquired by ribbon scanning using a Nikon 40x objective and a lateral per-pixel resolution of 363nm. Refer to methods in the primary text for information pertinant to sample preparation.
(TIF)

S2 Fig Two color sequential scanning reduces cross-talk between channels. A mouse was infected subcutaneously with VEEV TrD TaV-cherry (red) and at 72 hours post infection, fluorescent beads (green) were introduced into the vasculature by cardiac perfusion. The brain was harvested, sunk in 30% sucrose overnight, embedded in O.C.T. medium, and sectioned to 100μm at -30°C. Cut sections were thawed and floated in PBS for 10min then cleared by CUBIC R1 prior to imaging on the Caliber I.D. ribbon scanning confocal. Imaging of vasculature (green) and virus (red) was completed sequentially with the Olympus 25X, 1.05NA, water objective to a depth of 2.08mm with a lateral per pixel resolution of 365nm and z-steps of 0.76μm. (A) A partial horizontal section of brain demonstrates three regions of virus replication (arrows). A magnified region, indicated by the red box, displays the individual channels (B) 488-green and (C) 561-red which demonstrate limited cross-talk. Virus and vasculature can be clearly distinguished in the (D) merged image.
(TIF)

S1 Movie Alignment of optical sections in Z. A whole mouse brain was harvested from an untreated animal and cleared using CUBIC R1. The cleared brain was washed three times for 2 hours each at 37 degrees Celsius in PBS, 0.1% (v/v) TritonX-100, 0.5% BSA. The brain stained for 7 days (1:1000) in the same solution with anti-GFAP alexa-594 (2E1.E9, Biolegend). Following staining, the brain was washed at 37 degrees Celsius three times for 2 hours and then overnight. The following day the brain was immersed in a variation of CUBIC R2 for which the volume of triethanolamine (TEA) was replaced with dH2O (CUBIC R2 w/o TEA) until clear. The brain was mounted and imaged in CUBIC R2 w/o TEA. The sample was imaged with the Olympus 40x, 1.3NA, oil objective with a lateral per pixel resolution of 317nm.
(MP4)

S2 Movie 3D imaging of a virus infected brain at subcellular detail. 3D image of EEEV TaV-GFP (green) infected mouse brain at 96hpi cooresponding to the data shown in Fig 4. Refer to methods in the primary text for information pertinant to sample preparation.
(MPG)

S3 Movie Multicolor 3D imaging of a virus infected brain using an Olympus 25X, 1.05NA objective. 3D image of VEEV TrD Tav-cherry (red) infected mouse brain at 96hpi. Fluorescent beads (green) were introduced into the vasculature by cardiac perfusion. These data coorespond to those shown in Fig 5. The data were acquired using the ribbon scanning confocal method with the Nikon 20X, 0.95NA objective. Refer to methods in the primary text for information pertinant to sample preparation.
(MPG)

S4 Movie Multicolor 3D imaging of a virus infected brain using a Nikon 20X, 0.95NA objective. 3D image of VEEV TrD Tav-cherry (red) infected mouse brain at 96hpi. Fluorescent beads (green) were introduced into the vasculature by cardiac perfusion. The sample is the same as that shown in shown in Fig 5. The data were acquired using the ribbon scanning confocal method with the Nikon 20X, 0.95NA objective. Refer to methods in the primary text for information pertinant to sample preparation.
(MPG)

S5 Movie Multicolor 3D imaging of a virus infected brain using Nikon 20X, 0.45NA ELWD objective. 3D image of VEEV TrD Tav-cherry (red) infected mouse brain at 96hpi. Fluorescent beads (green) were introduced into the vasculature by cardiac perfusion. The sample is the same as that shown in Fig 5. The data were acquired using the ribbon scanning confocal method with the Nikon 20X, 0.45NA ELWD objective. Background on the green channel was subtracted after 3D reconstruction. Refer to methods in the primary text for information pertinant to sample preparation.
(MPG)

S6 Movie Multicolor confocal imaging of large brain volumes using a new Nikon 10X, 0.5NA long working distance objective. 3D image of WEEV McM TaV-GFP (green) infected mouse brain at 96hpi. Fluorescent beads (red) were introduced into the vasculature by cardiac perfusion. The data were acquired in a single imaging session, to a depth of 4.2mm using 2.28μm z-steps (1850 z-planes). The reconstruction represents approximately ¼ of a whole brain at a resolution of 1430nm per-pixel. Refer to methods in the primary text for information pertinant to sample preparation.
(MPG)
