# Supplementary figures and images for: Ribbon scanning confocal for high-speed high-resolution volume imaging of brain
Source: PLoS One. 2017 Jul 7;12(7):e0180486. doi: 10.1371/journal.pone.0180486 (PMC5501561; doi:10.1371/journal.pone.0180486)

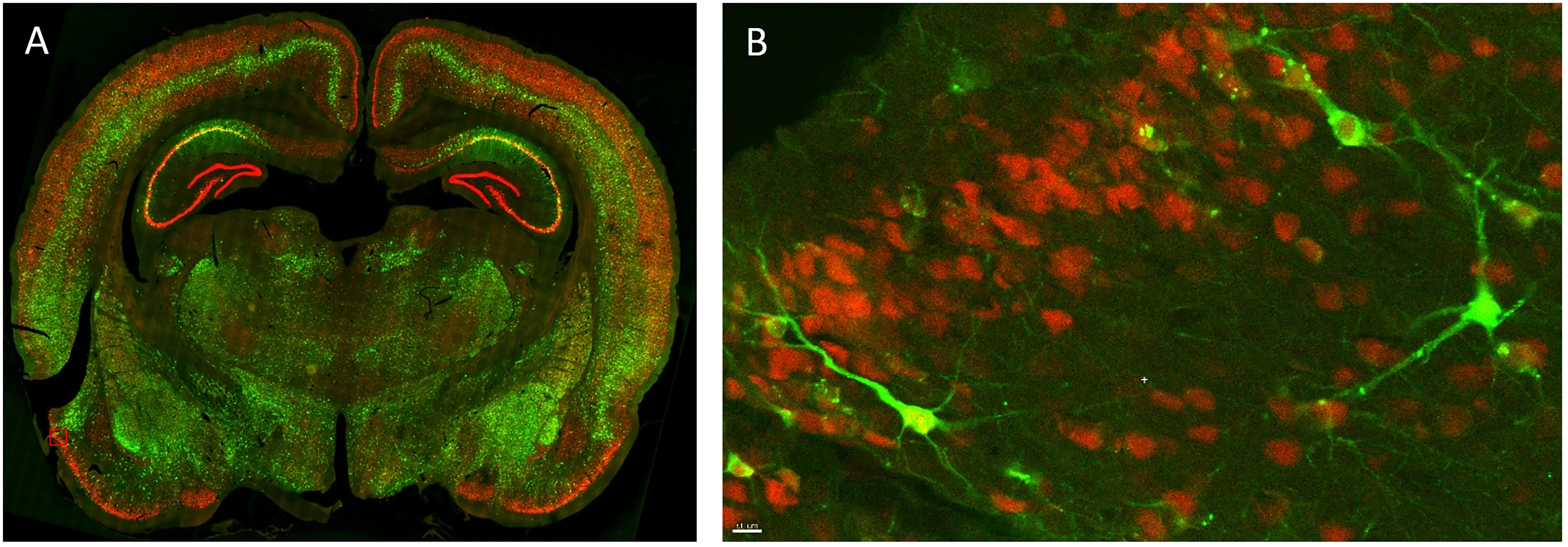

Supplement: S1 Fig — (A) A whole coronal section of a rabies infected rat brain was stained for rabies (green) and nuclei (NeuN, red). (B) A zoomed imaged from panel A with the location designated by the red box. The image was acquired by ribbon scanning using a Nikon 40x objective and a lateral per-pixel resolution of 363nm. Refer to methods in the primary text for information pertinant to sample preparation. (TIF) [file pone.0180486.s001.tif]

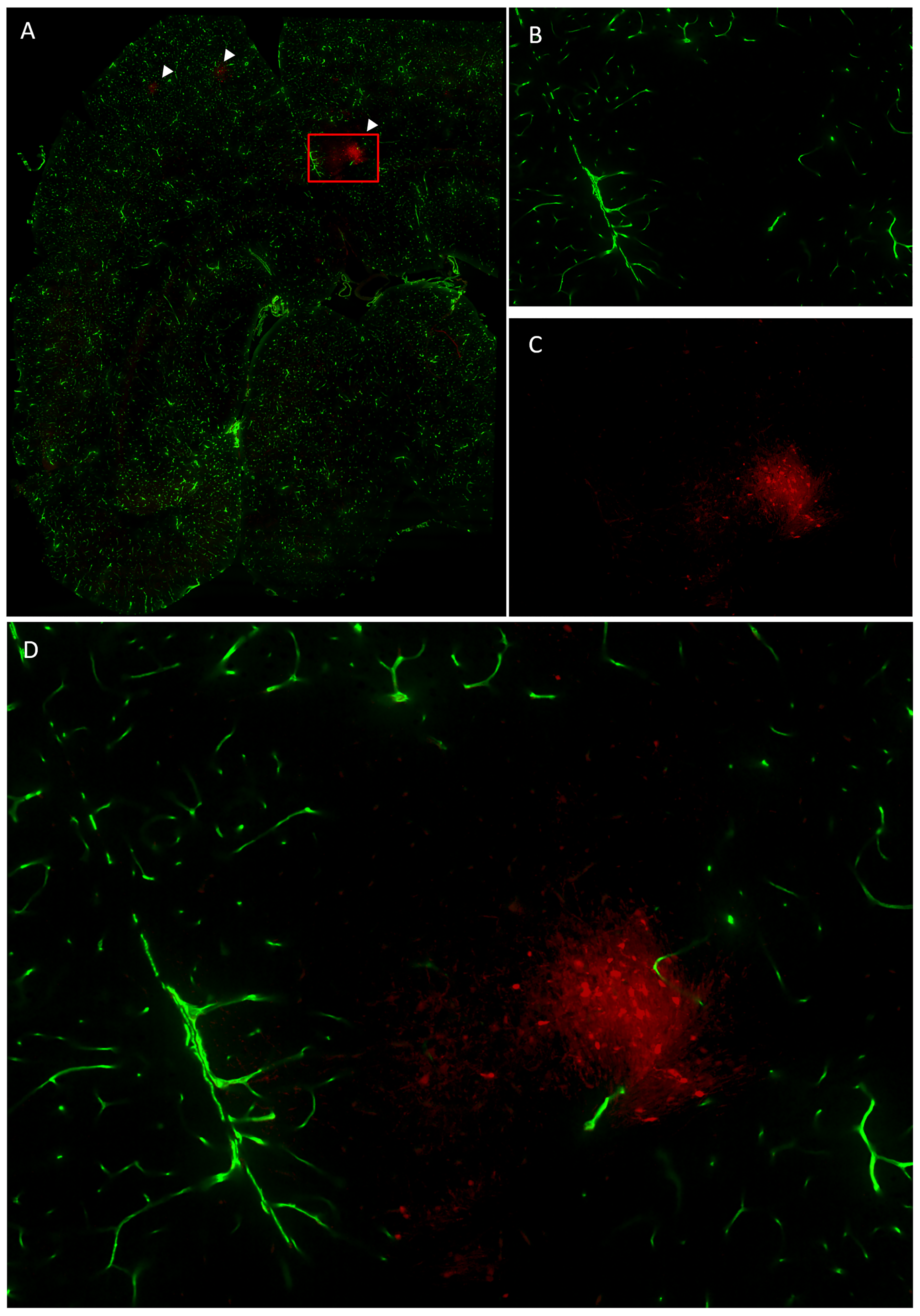

Supplement: S2 Fig — A mouse was infected subcutaneously with VEEV TrD TaV-cherry (red) and at 72 hours post infection, fluorescent beads (green) were introduced into the vasculature by cardiac perfusion. The brain was harvested, sunk in 30% sucrose overnight, embedded in O.C.T. medium, and sectioned to 100μm at -30°C. Cut sections were thawed and floated in PBS for 10min then cleared by CUBIC R1 prior to imaging on the Caliber I.D. ribbon scanning confocal. Imaging of vasculature (green) and virus (red) was completed sequentially with the Olympus 25X, 1.05NA, water objective to a depth of 2.08mm with a lateral per pixel resolution of 365nm and z-steps of 0.76μm. (A) A partial horizontal section of brain demonstrates three regions of virus replication (arrows). A magnified region, indicated by the red box, displays the individual channels (B) 488-green and (C) 561-red which demonstrate limited cross-talk. Virus and vasculature can be clearly distinguished in the (D) merged image. (TIF) [file pone.0180486.s002.tif]
